# Prognostic comparison between implantable cardioverter‐defibrillator and amiodarone in cancer patients

**DOI:** 10.1002/joa3.70093

**Published:** 2025-05-19

**Authors:** Hirota Kida, Toshitaka Morishima, Eiji Uza, Hironori Yamamoto, Taku Yasui, Masashi Fujita, Isao Miyashiro

**Affiliations:** ^1^ Department of Clinical Engineering Osaka International Cancer Institute Osaka Japan; ^2^ Cancer Control Center Osaka International Cancer Institute Osaka Japan; ^3^ Department of Onco‐Cardiology Osaka International Cancer Institute Osaka Japan

**Keywords:** amiodarone, cancer, implantable cardioverter defibrillator

## Abstract

**Background:**

Implantable cardioverter‐defibrillator (ICD) has been demonstrated to improve survival outcomes compared to amiodarone. However, this effectiveness in cancer patients remains unclear. Given the complexity of cardiovascular management in this population, including cancer stage considerations, we evaluated the relative effectiveness of ICD versus amiodarone in cancer patients.

**Methods and Results:**

We linked cancer registry data with administrative records to identify patients newly prescribed amiodarone or who underwent ICD implantation between 2010 and 2015 at 36 hospitals in Osaka Prefecture, Japan. Among 161,125 cancer patients, 339 met the inclusion criteria (amiodarone: *n* = 281; ICD: *n* = 58), with a median follow‐up of 762 days. Kaplan–Meier analysis revealed that the ICD group had a significantly reduced risk of all‐cause mortality compared to the Amiodarone group (Log‐rank test, *p* < .003). Multivariable Cox proportional hazard regression model showed that ICD was an independent prognostic factor (Hazard ratio: 0.47, 95% confidence interval: 0.29–0.79, *p* = .004). These results were confirmed in a propensity‐matched analysis. Among patients with cancer stage: in situ or localized, no significant difference in survival risk was observed between the ICD and Amiodarone groups, and ICD was not significantly associated with all‐cause death. Conversely, among patients with cancer stage: regional or distant, the ICD group had a significantly reduced risk of all‐cause death compared to the Amiodarone group, and ICD was an independent prognostic factor.

**Conclusion:**

In cancer patients, ICD may improve long‐term prognosis compared to amiodarone, especially in patients with advanced cancer stages.

## Graphical abstract

The effectiveness of implantable cardioverter‐defibrillator (ICD) versus amiodarone in cancer patients remains unclear. Using 2010–2015 cancer registry data from 36 hospitals in Osaka, Japan, we found that ICD significantly improves cancer patients' prognosis compared to amiodarone.

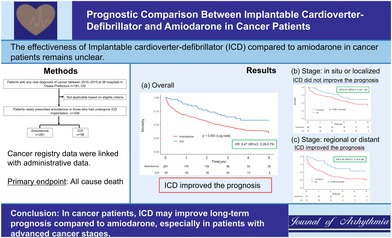

## 1 INTRODUCTION

Cancer constitutes a substantial proportion of global mortality,^1^ with projections indicating that over 28 million individuals will be living with cancer by 2040, representing a 47% rise compared to the 19.3 million cases reported in 2020.^2^ On the other hand, cancer mortality is declining^3^ and the long‐term survival rates among cancer patients have improved significantly on a global scale.^4^, ^5^ Cancer patients face a heightened risk of developing cardiovascular disease (CVD), as both diseases share overlapping risk factors such as hypertension, diabetes, and smoking.^6^ These comorbidities commonly coexist, and CVD remains the primary cause of noncancer‐related mortality among cancer patients.^7^

Implantable cardioverter‐defibrillator (ICD) is widely recognized as a highly effective treatment for the prevention of sudden cardiac death, supported by the evidence highlighting significant long‐term survival advantages in patients with the risk of ventricular arrhythmias.^8^, ^9^ Additionally, ICD is demonstrated to substantially improve survival outcomes compared to amiodarone.^10^ Conversely, cancer patients who underwent ICD implantation exhibit a poorer prognosis than their noncancer counterparts.^11^ Cardiovascular management for this population needs careful consideration of various factors, including the stage of cancer. Furthermore, the effectiveness of ICD compared to amiodarone in cancer patients remains unclear. In our study, we evaluated the relative effectiveness of ICD over amiodarone in cancer patients.

## 2 METHODS

### 2.1 Data sources

In this study, we integrated two distinct data sources to create a comprehensive and unified database for analyzing the association between patient mortality and clinical parameters, such as ICD implantation and the use of amiodarone, which are not typically recorded in cancer registries.^12^, ^13^, ^14^, ^15^ Data were retrieved from patients diagnosed with cancer between 2010 and 2015 for analysis. The primary data source was the Osaka Cancer Registry (OCR), a population‐based registry that systematically gathers detailed information on cancer diagnoses and outcomes for residents of Osaka Prefecture, Japan. OCR data include patient characteristics such as gender, age at cancer diagnosis, vital status, dates of death or the last follow‐up, cancer site, and cancer stage. The cancer stage were classified as in situ, localized, regional, distant, or unknown at diagnosis based on Surveillance, Epidemiology, and End Results (SEER).^16^ The definition of in situ stage is the presence of malignant cells within the cell group from which they arose with no penetration of the basement membrane of the tissue and no stromal invasion. The definition of localized stage is a malignancy limited to the organ of origin, which spread no farther. The definition of regional stage is tumor extension beyond the limits of the organ of origin. The definition of distant stage is that tumor cells that have broken away from the primary tumor, have traveled to other parts of the body, and have begun to grow at the new location. Vital status follow‐up was routinely conducted using death certificates. Additionally, in May 2019, official resident registries were employed to verify and update the vital status of patients.

The second data source comprised administrative records derived from Japan's Diagnosis Procedure Combination (DPC) Per‐Diem Payment System, which regulates insurer reimbursements to acute care hospitals. DPC data are among the most widely used hospital administrative datasets for research in Japan.^17^ These records provide clinical summaries and detailed insurance claims, including information on comorbidities, New York Heart Association (NYHA) classification, medications, and ICD implantation. In collaboration with the Council for Coordination of Designated Cancer Care Hospitals in Osaka, we obtained DPC data from hospitals affiliated with the OCR. The two datasets were merged at the patient level using each hospital's unique patient identification number as the linkage key.

### 2.2 Study population

We identified cancer from OCR regardless of the location. Cancers were grouped into the following 16 categories: mouth/pharynx (topographical codes: C00–14), esophagus (C15), stomach (C16), colorectal (C18–20), liver (C22), gallbladder and bile duct (C23–24), pancreas (C25), lung (C33–34), skin (C44), breast (C50), cervix uteri (C53), prostate (C61), kidney (C64), bladder (C67), urinary tract (C68), and other cancers (all other Cxx codes) based on the International Classification of Diseases for Oncology Third Edition (ICD‐O‐3). In instances of simultaneous multiple cancer diagnoses, the malignancy with the most advanced stage was designated as the primary cancer. We identified patients who were newly prescribed amiodarone or who underwent ICD implantation, including those receiving cardiac resynchronization therapy defibrillator (CRT‐D), following a cancer diagnosis between January 1, 2010 and December 31, 2015 from linked data. Patients who had prescribed amiodarone or who had undergone ICD implantation prior to their cancer diagnosis were excluded.

### 2.3 Comorbidities and medications

The structure of DPC data enables hospitals to document the presence of comorbidities for each patient. All comorbidities were extracted from the relevant data fields in the DPC file corresponding to the most recent hospitalization where amiodarone was prescribed or an ICD (include CRT‐D) was implanted. Comorbidities are documented utilizing the International Classification of Diseases, Tenth Revision (ICD‐10) codes. To identify a history of diabetes, chronic heart failure, chronic kidney disease, prior myocardial infarction, cardiomyopathy, and ventricular tachycardia/ventricular fibrillation (VT/VF), we classified the disease recorded on the DPC in accordance with QUAN's report^18^ and the guidelines of the Japanese Circulation Society.^19^, ^20^ The details of these classifications are shown in Table S1. Data on medications such as beta‐blockers, angiotensin‐converting enzyme inhibitors (ACEs), angiotensin II receptor blockers (ARBs), and mineralocorticoid receptor antagonists (MRAs) were extracted from the DPC database.

### 2.4 Endpoint

The primary endpoint of this study was all‐cause mortality. The outcomes were evaluated at 5 years after newly prescribed amiodarone or an ICD (include CRT‐D) was implanted. This study was approved by the local ethics committee of Osaka International Cancer Institute, which approved the study protocol (Approval number: 1707105108). Informed consent was not required, as the study was retrospective in nature, and the committee allowed for the opt‐out approach for the secondary use of existing data.

### 2.5 Statistical analysis

Categorical variables were presented as number (frequency) and compared using the chi‐squared test or Fisher's exact test between 2 groups, amiodarone group and ICD group. Continuous variables were presented as medians (interquartile) and compared using Mann–Whitney U test between 2 groups. Days of survival were analyzed with Kaplan–Meier curves and differences were tested using the log‐rank test between 2 groups. Multivariable COX proportional hazard model was used to assess the effect of ICD adjusted for the following clinically relevant covariates selected based on clinical consensus: age, gender, diabetes, chronic kidney disease, prior myocardial infarction, cardiomyopathy, NYHA class II or more, VT/VF, cancer stage: in situ or localized, interval from diagnosis of cancer to treatment, and medications such as β‐blockers, ACEs, ARBs, and MRAs.

Then, we divided patients into two groups based on the cancer stage: in situ or localized, and regional or distant. In each group, days of survival were analyzed with Kaplan–Meier curves and differences were tested using the log‐rank test. A multivariable COX proportional hazard model was used to assess the effect of ICD adjusted for the following clinically relevant covariates: age, gender, diabetes, chronic kidney disease, prior myocardial infarction, cardiomyopathy, VT/VF, and interval from diagnosis of cancer to treatment.

As a sensitivity analysis, we further evaluated the association between ICD implantation and prognosis within a propensity score‐matched cohort comprising patients who newly prescribed amiodarone or underwent ICD implantation. The propensity score for each patient was calculated using logistic regression incorporating the following parameters: age, gender, cancer stage: in situ or localized, cancer stage: regional, cancer stage: distant, interval from diagnosis of cancer to treatment, cardiomyopathy, VT/VF, mainly focusing on variables related to cancer. Using propensity scores, 1:1 nearest‐neighbor matching was conducted with a caliper width set at 0.20 times the standard deviation of the propensity score to match these patients. The matching process was implemented using the function Match from the “Matching” package in R. The proportional hazards assumption for ICD related to the primary endpoint was validated through the analysis of Schoenfeld residuals (*p* > .05). All analyses were performed using R software (version 4.0.0; R Foundation for Statistical Computing, Vienna, Austria) with R Studio (version 3,6,1; Boston, MA, USA), and two‐tailed *p* values <.05 were considered indicative of statistical significance.

## 3 RESULTS

### 3.1 Baseline characteristics

**FIGURE 1:**
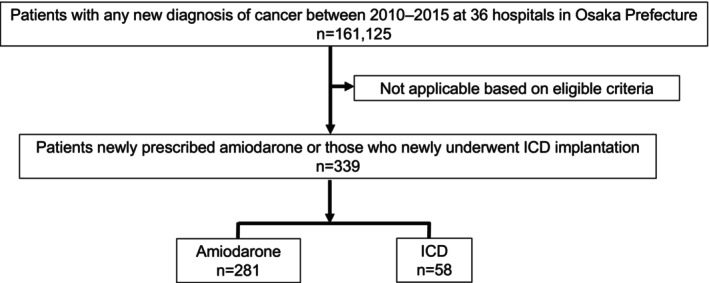
Study population. ICD, Implantable cardioverter‐defibrillator.

From a large consolidated database (*N* = 161.125), we identified 339 patients who were newly prescribed amiodarone (*N* = 281) or underwent ICD implantation (*N* = 58) (Figure 1). Baseline clinical characteristics are shown in Table 1. The ICD group included 38 of the 58 patients (65.5%) who underwent CRT‐D as well as ICD. The majority of patients in both the Amiodarone and ICD groups were predominantly at cancer stages: in situ or localized (the Amiodarone group: 59.8%, the ICD group: 63.8%). In both groups, the predominant sites of cancer occurrence, ranked in descending order, were the colon (the Amiodarone group: 19.9%, the ICD group: 20.7%), stomach (the Amiodarone group: 12.5%, the ICD group: 19.0%), lung (the Amiodarone group: 10.0%, the ICD group: 5.2%), and liver (the Amiodarone group: 7.5%, the ICD group: 5.2%). Compared to the Amiodarone group, the ICD group significantly had a higher prevalence of cardiomyopathy, chronic heart failure, VT/VF, NYHA II or more, and drugs such as ACEs or ARBs, MRAs, β‐blockers, and a longer interval between cancer diagnosis and either ICD implantation or amiodarone prescription.

**TABLE 1:** Baseline characteristics.

	Amiodarone	ICD	*p* value
Number	281	58	
Patient data			
Age, (years)	72 [66, 78]	70 [63, 75]	.084
Male, (%)	216 (76.9)	47 (81.0)	.603
CRT‐D, (%)	0	38 (65.5)	
Diabetes, (%)	80 (28.5)	24 (41.4)	.074
Chronic kidney disease, (%)	60 (21.4)	13 (22.4)	.997
Prior myocardial infarction	44 (15.7)	11 (19.0)	.670
Chronic heart failure, (%)	159 (56.6)	43 (74.1)	.020
Cardiomyopathy, (%)	25 (8.9)	16 (27.6)	<.001
NYHA II or more, (%)	45 (16.0)	21 (36.2)	.001
History of VT or VF, (%)	47 (16.7)	36 (62.1)	<.001
Cancer			
Interval between cancer diagnosis and Amiodarone or ICD (days)	298 [58, 955]	592 [301, 1307]	.004
Cancer stage: in situ or localized, (%)	168 (59.8)	37 (63.8)	.674
Cancer stage: regional, (%)	52 (18.5)	12 (20.7)	.714
Cancer stage: distant, (%)	30 (10.7)	4 (6.9)	.478
Stomach, (%)	35 (12.5)	11 (19.0)	.268
Colon, (%)	56 (19.9)	12 (20.7)	>.999
Lung, (%)	28 (10.0)	3 (5.2)	.367
Liver, (%)	21 (7.5)	3 (5.2)	.733
Medications			
ACE or ARB, (%)	180 (64.1)	46 (79.3)	.037
MRA, (%)	132 (47.0)	41 (70.7)	.002
β‐blocker, (%)	193 (68.7)	53 (91.4)	.001

*Note*: Data are expressed as median [interquartile range] or number (column percentage).

Abbreviations: ACE, angiotensin‐converting enzyme inhibitor; ARB, angiotensin II receptor blockers; CRT‐D, cardiac resynchronization therapy defibrillator; ICD, Implantable cardioverter‐defibrillator; MRA, mineralocorticoid receptor antagonist; NYHA, New York Heart Association; VF, ventricular fibrillation; VT, ventricular tachycardia.

### 3.2 Clinical endpoint

**FIGURE 2:**
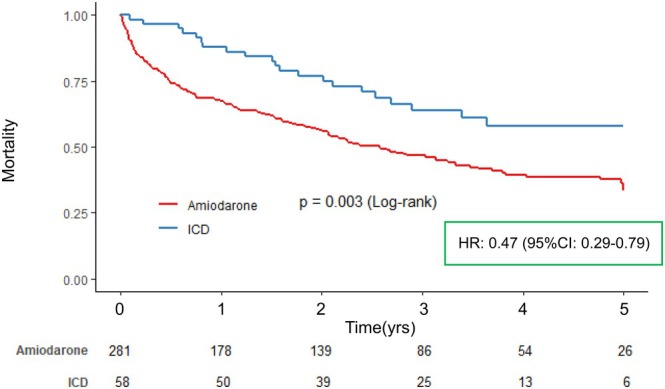
Kaplan–Meier curves stratified for 5‐year all‐cause death. We used a multivariable Cox proportional hazard model to compare the impact of ICD with Amiodarone on long‐term all‐cause mortality (5 years). Multivariable model: Adjusted for age, gender, diabetes, chronic kidney disease, prior myocardial infarction, cardiomyopathy, NYHA class II or more, VT/VF, cancer stage: In situ or localized, interval from diagnosis of cancer to treatment, and medications such as β‐blockers, ACEs, ARBs, and MRAs. ACEs, angiotensin‐converting enzyme inhibitors; ARBs, angiotensin II receptor blockers; CI, confidence interval; HR, hazard ratio; ICD, Implantable cardioverter‐defibrillator; MRAs, mineralocorticoid receptor antagonists; NYHA, New York Heart Association; VF, ventricular fibrillation; VT, ventricular tachycardia.

During a median follow‐up period of 762 days, among a total 339 patients, 179 patients (52.8%) died within 5 years after diagnosis of cancer. Kaplan–Meier analysis revealed that the ICD group had a significantly reduced risk of all‐cause death than the Amiodaron group, with a 5‐year survival rate of 58.0% versus 33.4%, respectively (Log‐rank test, *p* < .003) (Figure 2). Multivariable Cox proportional hazard regression model showed that ICD was an independent prognostic factor (Hazard ratio: 0.47, 95% confidence interval: 0.29–0.79, *p* = .004).

**FIGURE 3:**
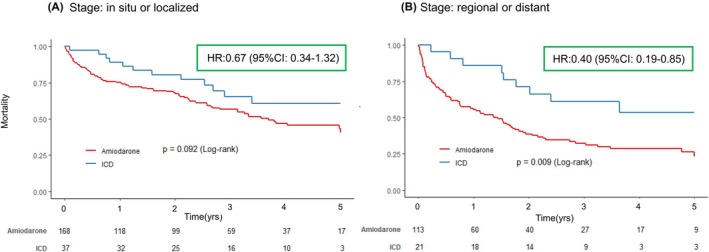
Kaplan–Meier curves stratified by cancer stages (stage: In situ or localized and stage: Regional or distant). Panel (A) indicates Kaplan–Meier curves for cancer stage: In situ or localized. Panel (B) indicates Kaplan–Meier curves for cancer stage: Regional or distant. We used a multivariable Cox proportional hazard model to compare the impact of ICD with Amiodarone on long‐term all‐cause mortality (5 years). Multivariable model: Adjusted for age, gender, diabetes, chronic kidney disease, prior myocardial infarction, chronic heart failure, VT/VF, interval from diagnosis of cancer to treatment. CI, confidence interval; HR, hazard ratio; ICD, Implantable cardioverter‐defibrillator; VF, ventricular fibrillation; VT, ventricular tachycardia.

Among patients with cancer stage: in situ or localized (*N* = 205), there were 91 all‐cause deaths (44.4%) over a median follow‐up period of 867 [304, 1398] days. Kaplan–Meier analysis indicated no significant difference in survival risk between the ICD and Amiodarone groups, with 5‐year survival rates of 60.7% and 40.4%, respectively (Log‐rank test, *p* = .092). In the multivariable Cox proportional hazard regression model, ICD was not significantly associated with all‐cause death (Hazard ratio: 0.67, 95% confidence interval: 0.34–1.32, *p* = .252). (Figure 3A). Conversely, among patients with cancer stage: regional or distant (*N* = 134), there were 88 all‐cause deaths (65.7%) over a median follow‐up period of 563 [121, 1172] days. Kaplan–Meier analysis revealed that the ICD group had a significantly reduced risk of all‐cause death compared to the Amiodarone group, with 5‐year survival rates of 53.6% and 23.2%, respectively (Log‐rank test, *p* = .009). The multivariable Cox proportional hazard regression model showed that ICD was an independent prognostic factor (Hazard ratio: 0.40, 95% confidence interval: 0.19–0.85, *p* = .017). (Figure 3B).

### 3.3 Sensitivity analysis

**FIGURE 4:**
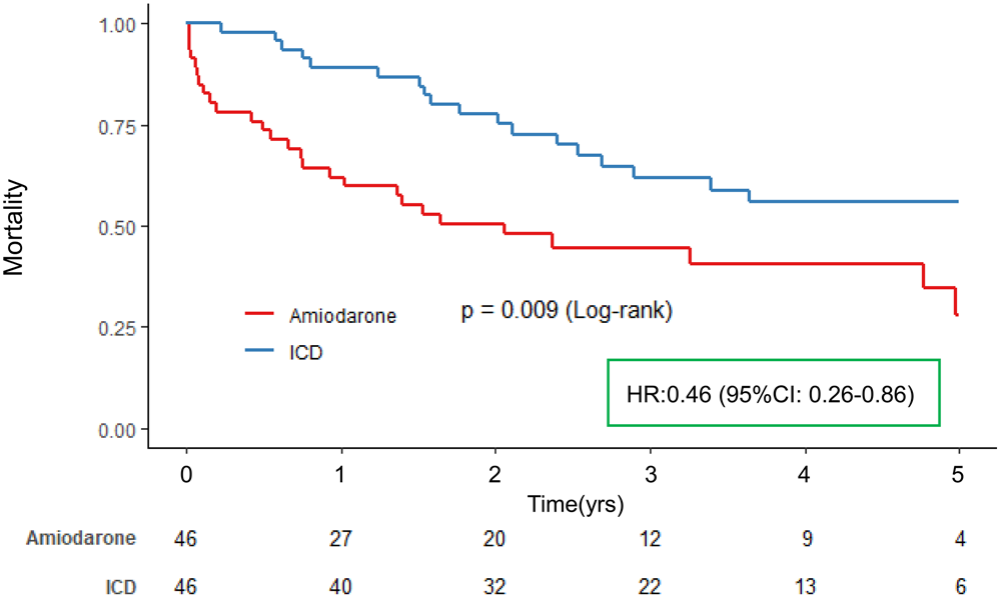
Kaplan–Meier curves after propensity score matching. Propensity score was estimated for each patient by logistic regression using the following parameters: age, gender, cancer stage: In situ or localized, cancer stage: Regional, cancer stage: Distant, interval from diagnosis of cancer to treatment, cardiomyopathy, VT/VF. Based on the propensity score, 1:1 matching was performed between patients with Amiodarone and ICD. We used a univariable Cox proportional hazard model to compare the impact of ICD with Amiodarone on long‐term all‐cause mortality (5 years). CI, confidence interval; HR, hazard ratio; ICD, Implantable cardioverter‐defibrillator; VF, ventricular fibrillation; VT, ventricular tachycardia.

Propensity score matching identified 92 patients between the ICD group and the Amiodarone group (46 patients each) (Table 2). Baseline characteristics were generally well balanced and were largely comparable between the two groups (Table 2). Kaplan–Meier analysis and univariable Cox proportional hazard model demonstrated significantly better 5‐year all‐cause outcomes in the ICD group compared to the Amiodarone group (Log‐rank test, *p* = .009; Hazard ratio: 0.46, 95% confidence interval: 0.26–0.81, *p* = .008) (Figure 4).

**TABLE 2:** Baseline characteristics after propensity score matching.

	Amiodarone	ICD	*p* value	SMD
Number	46	46		
Patient data				
Age, (years)	70 [65, 78]	73 [67, 76]	.907	0.083
Male, (%)	11 (23.9)	8 (17.4)	.606	0.162
Diabetes, (%)	15 (32.6)	20 (43.5)	.39	0.225
Chronic kidney disease, (%)	8 (17.4)	11 (23.9)	.606	0.162
Prior myocardial infarction	9 (19.6)	10 (21.7)	>.999	0.054
Cardiomyopathy, (%)	12 (26.1)	11 (23.9)	>.999	0.050
Chronic heart failure, (%)	35 (76.1)	34 (73.9)	>.999	0.050
NYHA II or more, (%)	11 (23.9)	17 (37.0)	.257	0.286
History of VT or VF, (%)	24 (52.2)	24 (52.2)	>.999	<0.001
Cancer				
Interval between cancer diagnosis and treatment (days)	487 [66, 1058]	530 [273, 921]	.517	0.096
Cancer stage: in situ or localized, (%)	25 (54.3)	27 (58.7)	.833	0.088
Cancer stage: regional, (%)	14 (30.4)	10 (21.7)	.476	0.199
Cancer stage: distant, (%)	4 (8.7)	4 (8.7)	>.999	<0.001
Stomach, (%)	3 (6.5)	8 (17.4)	.199	0.34
Colon, (%)	12 (26.1)	10 (21.7)	.807	0.102
Lung, (%)	5 (10.9)	3 (6.5)	.711	0.155
Liver, (%)	1 (2.2)	0 (0.0)	>.999	0.211
Medications				
ACE or ARB, (%)	33 (71.7)	38 (82.6)	.32	0.261
MRA, (%)	27 (58.7)	34 (73.9)	.186	0.326
β‐blocker, (%)	36 (78.3)	41 (89.1)	.259	0.297

*Note*: Data are expressed as median [interquartile range] or number (column percentage).

Abbreviations: SMD, standardized mean difference; others are the same as Table 1.

## 4 DISCUSSION

The present study from cancer registry linked with administrative data demonstrated the following findings: (1) cancer patients with ICDs had a better long‐term outcome than those with amiodarone, and ICD was an independent prognostic factor among this population; (2) in patients with cancer stage: in situ or localized, ICDs showed no significant association with long‐term outcome; and (3) in patients with cancer stage: regional or distant, ICD was an independent prognostic factor.

In cancer patients, relative benefit of ICD therapy might be limited because of competing risk of nonarrhythmic mortality such as cancer death. Research on ICD therapy limited to cancer patients might be difficult because of sample size, so there is no information on comparing the long‐term prognosis between patients with ICD and amiodarone in cancer patients. However, using large‐scale cancer registry linked with administrative data, our study is the first to demonstrate that in cancer patients, ICD therapy might improve long‐term outcome compared to amiodarone. Enriquez et al. retrospectively analyzed 1598 patients who received an ICD implantation at two facilities in Ontario, Canada, between 2007 and 2015. Of these, 209 (13.1%) were diagnosed with cancer, and the occurrence of ventricular arrhythmia was significantly higher than in patients without cancer.^21^ In a cohort study for patients with a first‐time ICD implantation (*N* = 5665) using Danish nationwide registry from 2007 to 2012, out of these [289 (5.1%)] were diagnosed with cancer, and cancer was associated with elevated mortality in patients with ICD for secondary prevention, whereas no such association was observed in those for primary prevention.^11^ Since both studies were conducted on patients with pre‐implanted ICD, the ability to comprehensively assess the efficacy of ICD in cancer patients remains limited. On the other hand, in a single‐center retrospective cohort study for patients with cancer who were eligible for primary ICD therapy, there was no statistically significant difference in survival rates between cancer patients with and without ICD (hazard ratio 0.521, *p* = .127).^22^ However, this study was constrained by a limited sample size (*N* = 75), and the impact of antiarrhythmic drugs such as amiodarone, was not assessed.

The strength of our study stems from using a large‐scale cancer registry linked with administrative data, which enabled us to identify patients who received a new ICD implantation or were newly prescribed amiodarone after a cancer diagnosis. Among cancer patients, ICD showed a significant impact on improving prognosis compared to amiodarone. This result was also consistent in the sensitivity analysis using propensity score matching. Notably, among patients with cancer stage: in situ or localized, ICD demonstrated no significant advantage in prognosis compared to amiodarone; nevertheless, among patients with a more advanced cancer stage: regional or distant, ICD was associated with a significant improvement in prognosis compared to amiodarone. As elaborated upon subsequently, the progression of cancer may elevate the risk of ventricular arrhythmias. With the global rise in cancer prevalence and improved long‐term survival rates,^2^, ^4^, ^5^ healthcare providers are increasingly encountering complex clinical scenarios, such as choosing between ICD or amiodarone for cancer patients, necessitating more nuanced decision‐making in this situation. However, any guidelines have no mention of the relationship between ICD implantation and cancer and recommend a minimum estimated survival of 1 year.^9^, ^20^, ^23^ Only 27% of patients eligible for a primary prevention ICD, as per guideline recommendations, ultimately received the device.^22^ Therefore, it is crucial to assess the suitability of ICD implantation within this population, and our findings may provide valuable insights toward this problem, though further research is needed to optimize their application in the oncologic setting.

### 4.1 Heart disease in cancer patients

In a cohort study that used data from the same registry, cancer patients had a higher risk of cardiac death than general patients, and the standardized mortality ratio for heart disease was 2.80 (95% CI, 2.74–2.85), and the risk of heart‐related death showed a progressive increase over time following a cancer diagnosis.^7^ Inflammation enhances all stages of carcinogenesis, and Neuroendocrine factors, oxidative stress, pro‐inflammatory cytokines, and dysregulation of the immune system have been identified as contributors to the heightened risk of cardiovascular disease in oncology patients.^6^, ^24^ Certain anticancer agents, such as anthracyclines,^25^ tyrosine kinase inhibitors,^26^ and immune checkpoint inhibitors,^27^, ^28^ are associated with cardiotoxicity as an adverse side effect. Numerous other anticancer agents have been documented to possess the potential to induce arrhythmias.^29^ Moreover, an increased risk of cardiovascular complications linked to surgical interventions and radiotherapy has also been reported.^30^, ^31^ Therefore, cancer patients are generally considered a population at an increased risk for adverse cardiac events. In our cohort, chronic heart failure was prevalent in 59.3% of patients (201 out of 339), indicating that this group of cancer patients faced an exceptionally increased risk of adverse cardiac events, including VT/VF. Nonsustained VT and frequent premature ventricular contractions in 24‐h electrocardiogram recording were factors that worsened the long‐term prognosis among cancer patients.^32^, ^33^ From the above, understanding the intricate relationship between cancer therapies and arrhythmogenesis is crucial for devising targeted interventions, such as ICD or amiodarone, aimed at mitigating the prevalence of arrhythmic events in this population.

### 4.2 Cancer progression and ventricular arrhythmias

In our study, among patients with cancer stage: in situ or localized, ICD demonstrated no significant advantage in prognosis compared to amiodarone. On the other hand, among patients with more advanced cancer stage: regional or distant, ICD was associated with a significant improvement in prognosis compared to amiodarone. In a previous study for cancer patients with ICD, patients with stage 4 had a significantly more frequent incidence of VT/VF than those with other stages.^21^ Curative treatment is typically the primary strategy for early‐stage cancer, often supplemented by adjuvant chemotherapy. On the other hand, in advanced cancer, molecular‐targeted therapies and immune checkpoint inhibitors frequently constitute the cornerstone of treatment alongside conventional anticancer agents. However, it is well established that these anticancer drugs exhibit a proarrhythmic effect, including prolongation of the QT interval.^29^ The risk of heart‐related death showed a progressive increase over time following a cancer diagnosis.^7^ Therefore, it is evident that patients with advanced stages of cancer face a heightened risk of fatal arrhythmias, suggesting a potential increase in the effectiveness of ICD within this population. Considering the improvement in the long‐term prognosis for cancer patients,^4^, ^5^ it may be essential to conduct individualized assessments of prognosis, even for those with advanced stages of cancer, and to make decisions from multiple perspectives.

### 4.3 Limitations

Our study has several limitations. First, the dosage of amiodarone administered could not be identified because of the unavailability of information from the linked dataset. The dosage of amiodarone can significantly influence the suppression of VT/VF, representing a major limitation. Second, though the ICD group included 65.5% who underwent CRT‐D, we could not evaluate the impact of CRT‐D through biventricular pacing on improving outcomes. Third, despite performing multivariable analyses and propensity score matching, the potential for residual selection bias in the allocation of ICD or amiodarone might not be entirely eliminated. Fourth, because of the unavailability of blood and echocardiographic data, we were unable to include these parameters in our evaluation. Fifth, the specific cause of death, such as cancer or cardiovascular disease, could not be identified from our dataset. Finally, amiodarone exerts a QT interval‐prolonging effect, and it cannot be ruled out that this property may have contributed to an increased risk of proarrhythmic events in cancer patients.

## 5 CONCLUSIONS

In cancer patients, ICD may improve long‐term prognosis compared to amiodarone, especially in patients with advanced cancer stages.

## AUTHOR CONTRIBUTIONS

I.M. contributed to the acquisition of data. T.M. contributed to the curation and interpretation of data. All authors revised the manuscript critically and approved the final version of the manuscript. All authors agree to be accountable for all aspects of the work in ensuring that questions related to the accuracy or integrity of any part of the work are appropriately investigated and resolved.

## CONFLICT OF INTEREST STATEMENT

There is no conflict of interest to declare.

## ETHICS APPROVAL STATEMENT

This study was approved by the local ethics committee of Osaka International Cancer Institute, which approved the study protocol (Approval number: 1707105108). Informed consent was not required, as the study was retrospective in nature, and the committee allowed for the opt‐out approach for the secondary use of existing data.

## Supporting information


Data S1.

## Data Availability

The data that support the findings of this study are available on reasonable request from the corresponding author. The data are not publicly available because of privacy and ethical restrictions.
