# Supplementary material for: Prognostic comparison between implantable cardioverter‐defibrillator and amiodarone in cancer patients
Source: J Arrhythm. 2025 May 19;41(3):e70093. doi: 10.1002/joa3.70093 (PMC12086514; doi:10.1002/joa3.70093)
Supplement: Supplementary file 1 — Data S1. [file JOA3-41-e70093-s001.docx]

**Online appendix**

**Prognostic Comparison Between Implantable Cardioverter-Defibrillator and Amiodarone in Cancer Patients.**

**Supplemental Figure 1. Kaplan-Meier curves for 5-year all-cause death stratified by 3 groups (CRT-D, ICD, and amiodarone group)**


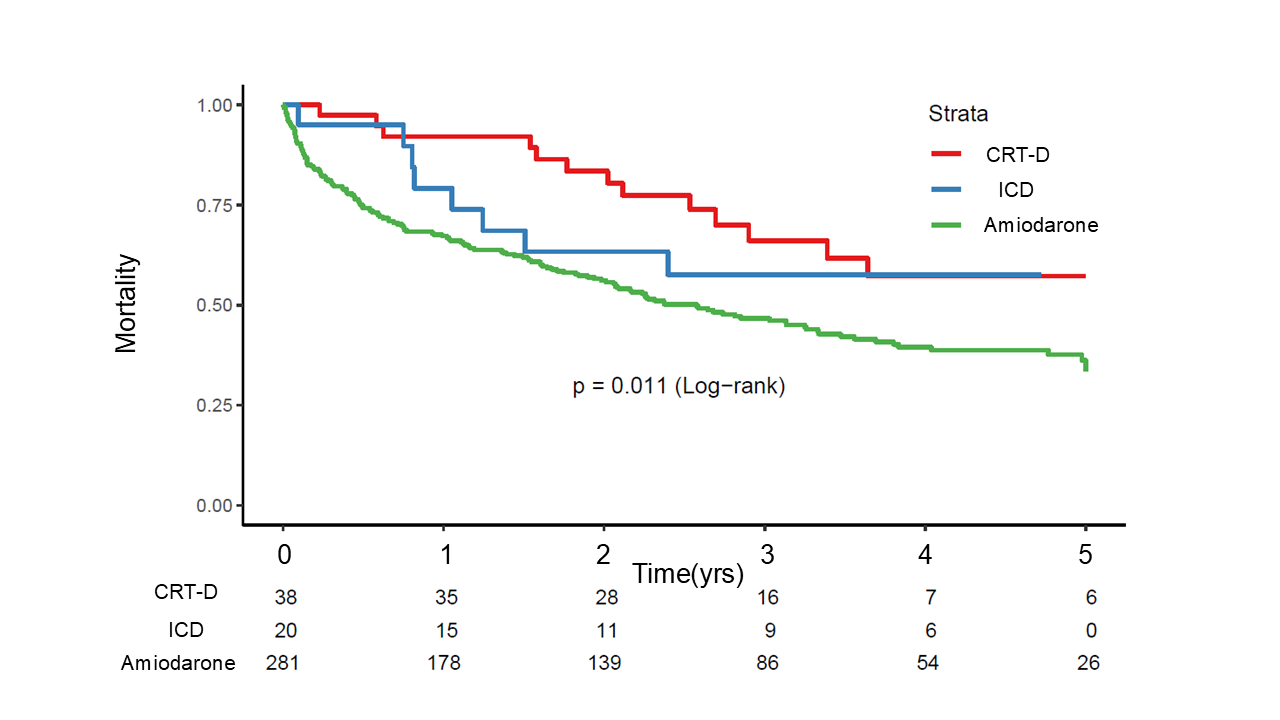


Kaplan-Meier analysis revealed that differences in overall survival among the three cohorts.

Abbreviations: CRT-D, cardiac resynchronization therapy-defibrillator; ICD, implantable cardioverter-defibrillator.

**Supplemental Table 1. Classification of diseases recorded in the Diagnosis Procedure Combination into various medical histories**

| DM | Chronic HF | CKD | Prior MI | Cardiomyopathy | VT / VF |
| --- | --- | --- | --- | --- | --- |
| ICD-10 code:  Disease name | ICD-10 code:  Disease name | ICD-10 code:  Disease or treatment name | ICD-10 code:  Disease name | Disease name | Disease name |
| E10: Insulin dependent DM | I09.9: Rheumatic HF | I12,13: Hypertensive Renal Disease | I121,122: MI | Hypertrophic cardiomyopathy | VF |
| E11: Non-insulin dependent DM | I11, I113: Hypertensive HF | N032-7: Chronic nephritis | I125.2: Old MI | Dilated cardiomyopathy | Sustained VT |
| E12-14: Other DM | I50: HF | N052-7: Chronic glomerulonephritis |  | Ischemic cardiomyopathy |  |
|  |  | N18,19: Chronic renal failure |  | ARVC |  |
|  |  | Z49: Maintenance dialysis |  |  |  |
|  |  | Z94: Renal transplantation |  |  |  |

Abbreviations: DM, diabetes mellitus; HF, heart failure; CKD, chronic kidney disease; MI, myocardial infarction; VT, ventricular tachycardia; VF, ventricular fibrillation; ICD-10, international classification of diseases, 10th revision; ARVC, arrhythmogenic right ventricular cardiomyopathy.
